# Checklists for Complications During Systemic Cancer Treatment Shared by Patients, Friends, and Health Care Professionals: Prospective Interventional Cohort Study

**DOI:** 10.2196/19225

**Published:** 2020-09-25

**Authors:** Helen V Jones, Harry Smith, Tim Cooksley, Philippa Jones, Toby Woolley, Derick Gwyn Murdoch, Dafydd Thomas, Betty Foster, Valerie Wakefield, Pasquale Innominato, Anna Mullard, Niladri Ghosal, Christian Subbe

**Affiliations:** 1 Ysbyty Gwynedd Penrhosgarnedd Bangor United Kingdom; 2 School of Medicine Cardiff Univeristy Cardiff United Kingdom; 3 The Christie Manchester United Kingdom; 4 UK Oncology Nursing Society Marlow United Kingdom; 5 Galactig Lôn Cae Ffynnon Cibyn Caernarfon United Kingdom; 6 North Wales Cancer Forum Bangor United Kingdom; 7 Cancer Chronotherapy Team Warwick Medical School Coventry United Kingdom; 8 European Laboratory U935, Institut National de la Santé et de la Recherche Médicale (INSERM) Paris-Saclay University Villejuif France; 9 North Wales Cancer Centre Rhyl United Kingdom; 10 School of Medical Sciences Bangor University Bangor United Kingdom

**Keywords:** cancer, patient safety, checklist, quality of life, anxiety, depression, health economics, mHealth, smartphone, redundancy

## Abstract

**Background:**

Advances in cancer management have been associated with an increased incidence of emergency presentations with disease- or treatment-related complications.

**Objective:**

This study aimed to measure the ability of patients and members of their social network to complete checklists for complications of systemic treatment for cancer and examine the impact on patient-centered and health-economic outcomes.

**Methods:**

A prospective interventional cohort study was performed to assess the impact of a smartphone app used by patients undergoing systemic cancer therapy and members of their network to monitor for common complications. The app was used by patients, a nominated “safety buddy,” and acute oncology services. The control group was made up of patients from the same institution. Measures were based on process (completion of checklists over 60 days), patient experience outcomes (Hospital Anxiety and Depression Scale and the General version of the Functional Assessment of Cancer Therapy at baseline, 1 month, and 2 months) and health-economic outcomes (usage of appointments in primary care and elective and unscheduled hospital admissions).

**Results:**

At the conclusion of the study, 50 patients had completed 2882 checklists, and their 50 “safety buddies” had completed 318 checklists. Near daily usage was maintained over the 60-day study period. When compared to a cohort of 50 patients with matching disease profiles from the same institution, patients in the intervention group had comparable changes in Hospital Anxiety and Depression Scale and General version of the Functional Assessment of Cancer Therapy. Patients in the Intervention Group required a third (32 vs 97 nights) of the hospital days with overnight stay compared to patients in the Control Group, though the difference was not significant. The question, “I feel safer with the checklist,” received a mean score of 4.27 (SD 0.87) on a Likert scale (1-5) for patients and 4.55 (SD 0.65) for family and friends.

**Conclusions:**

Patients undergoing treatment for cancer and their close contacts can complete checklists for common complications of systemic treatments and take an active role in systems supporting their own safety. A larger sample size will be needed to assess the impact on clinical outcomes and health economics.

## Introduction

Advances in cancer management continue to improve patient outcomes but are also associated with an increase in emergency presentations with disease- or treatment-related complications [1]. The challenges of acute oncology presentations have led to an interest in developing optimal care models and support systems for meeting patients’ needs [2]. Cancer patients seeking emergency care generally have longer lengths of stay, higher admission rates, and higher mortality than non-cancer patients [3].

Individualized management of acute cancer presentations is important to ensure services can mirror routine cancer care [4]. There is an increasing number of acute cancer presentations that can be risk-assessed for care in an outpatient ambulatory setting utilizing technology to support clinicians and patients. Complications of cancer and its treatments are predictable (fever, diarrhea, skin reactions, and drug-specific effects) and, in part, preventable.

Patients, friends, family, and other carers are often able to identify deviations from a patient’s normal status as a first step to facilitate calls for help. Peer support has been used in other settings to improve clinical care and safety, allowing families and friends to look after vulnerable patients, including those discharged after a stroke [5]. Mobile health apps for patients with cancer have the potential to track deterioration [6], support education, and recovery [7-9].

Modular redundancy is the duplication of critical components of a system to increase the reliability of performance in the design of technology [10] or clinical services [11]. Checklists allow redundancy by allowing multiple users to verify safety and are widely used in health care [12-15]. The United Kingdom Oncology Nursing Society (UKONS) has developed checklists for symptom-driven telephone triage [16].

Patients are competent to carry out surveillance and management of chronic conditions, as demonstrated by people with diabetes checking their blood sugars, people with asthma monitoring their peak flows, and people with heart failure recording their weight. Patients admitted to the hospital as medical emergencies can assist in the recording of key safety-critical information [17]. Information about cancer improves compliance, especially if it is tailored to individual needs and context-specific [4].

The study aimed to test the feasibility of a smartphone-based checklist that allows redundant access to safety-critical processes for patients, members of their immediate social network, and health care professionals to stimulate patients and carers to seek medical assistance when necessary while providing reassurance when appropriate.

## Methods

### Study Design

The trial was designed as a prospective interventional cohort study.

### Participants

Oncology patients attending outpatient clinics at the Ysbyty Gwynedd, North Wales, were invited to take part in the study. Patients were eligible if they had a known malignancy and were receiving treatment for cancer, including chemotherapy, radiotherapy, immunotherapy, or best supportive care. Patients were eligible for enrollment during the entirety of their treatment course.

Patients were excluded from the trial if they were receiving end of life care or lacked a smartphone to access the app. There were 50 app licenses available for the trial, and 100 patients were recruited. Patients who did not want to use the smartphone app and patients recruited after all the licenses had been distributed were recruited into a control group to provide indicative data on service usage in patients not using the app.

All participants, including controls, gave written informed consent.

### Smartphone App

**Figure 1:**
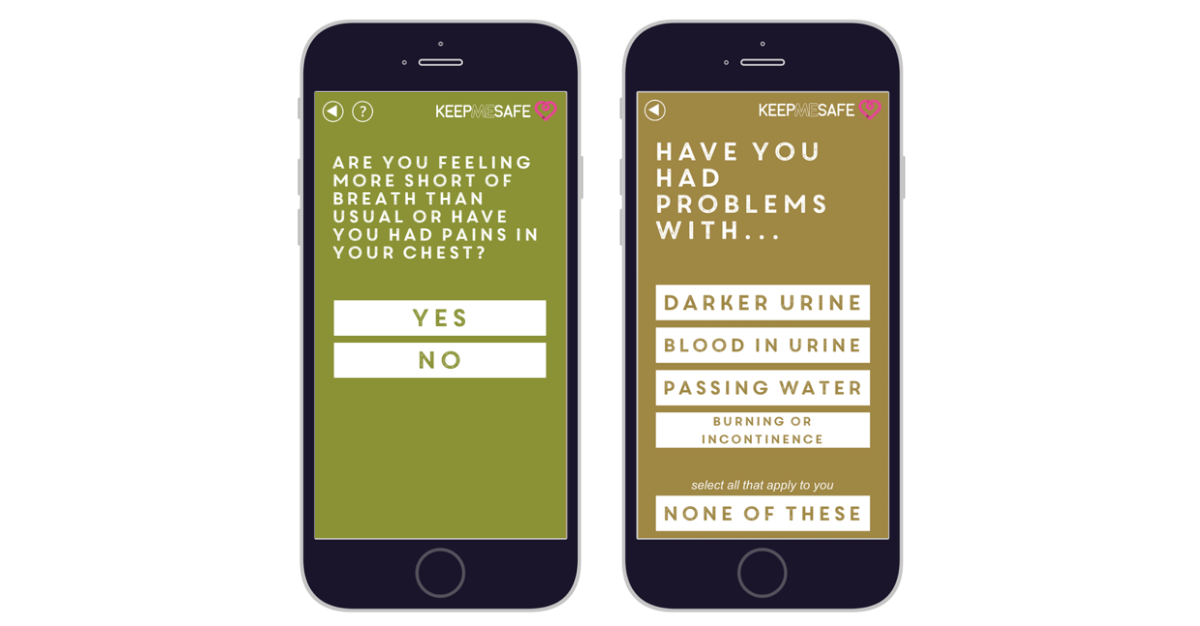
Sample screenshots of checklist items. Item 2 (breathlessness or chest pain) is linked to a red escalation, item 7 (urine problems) is linked to amber escalation.

The content of the app was coproduced in four focus group events. Focus groups consisted of 15 patient representatives, clinicians, and health-service researchers. Checklists were based on the UKONS 24-hour triage tool [16], the UKONS Oncology/Hematology risk assessment tool for Primary Healthcare Professionals [18], and a symptom assessment tool included in the Cancer Research UK Patient treatment record adapted from the UKONS 24-Hour Triage Tool [19]. UKONS tools classify symptoms and signs according to risk and urgency into green, amber, and red with linked actions for escalation from generic advice (green) to encouragement to seek a routine appointment or an urgent assessment (amber or red) (Figure 1).

Clinicians, patients, and researchers devised a hierarchy of safety-relevant symptoms in two iterations. Items were summarized in nine screens, and item rankings were decided by consensus. Checks were presented in order of priority, starting with the most urgent and life-threatening symptoms.

The system allows the addition of disease- or treatment-specific checks in the content management system. For this study, only a generic checklist was activated. Prototypes were tested against typical case studies. Symptoms that were “red flags” generated a recommendation to the patient to seek medical care. The app sent text reminders to patients once a day to complete the checklist.

**Figure 2:**
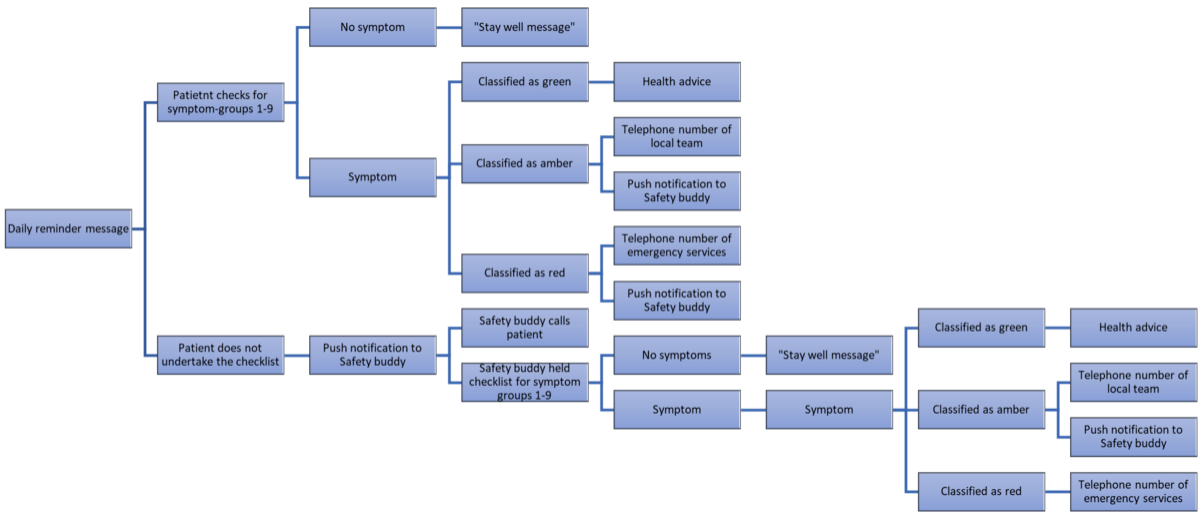
Workflow of the application.

Each patient was asked to invite one family member or friend to be their “Safety buddy.” Safety buddies also downloaded the checklist app to their smartphone. Safety buddies received push notifications if the patient did not complete the checklist within an agreed timeframe or if patients reported potentially serious symptoms (equivalent to red fields in the UKONS checklist): “You might want to call your friend/family member.” Safety buddies were then asked to complete the checklist on their phone with the patient (Figure 2).

A dashboard for the acute oncology team showed notifications and alerts that could be annotated by clinicians. Nurse specialists reviewed the reported symptoms once daily via an online dashboard and followed up with patients if the symptoms required further attention.

### Use of the App

Patients were enrolled for 60 days. Patients and the friend or family member were encouraged to access the app to record symptoms at least once daily. It was emphasized to patients that nursing staff would not be monitoring the app constantly and, therefore, the onus was on them to seek medical care if urged to do so by the app. App users received a call after a week to check for technical difficulties in app usage.

### Outcome Measures

The Hospital Anxiety and Depression Scale (HADS) [20] and the General version of the Functional Assessment of Cancer Therapy (FACT-G) [21] were completed at baseline, one month, and two months.

Health-economic outcomes consisted of the usage of appointments in primary care and elective and unscheduled hospital admissions.

### Patient Feedback

Patients and carers were able to provide feedback within the application. They were asked to use a Likert scale with gradings from 1 (strongly disagree) to 5 (strongly agree).

### Project Governance

The study was conducted according to the principles of the World Medical Association’s Declaration of Helsinki 2013 [22]. A study board supervised the development, testing, and evaluation. The group met every three months to issue interim reports and to review risk logs and possible adverse events. Ethics approval was granted (REC reference: 18/WA/0213).

## Results

### Recruitment

Patients were recruited from January 24, 2019, to September 17, 2019. Of the 197 patients approached, 100 agreed to participate—50 in the control group and 50 in the intervention group. Of the 100 participants, 56 were female. Groups were matched for gender, type of cancer, and performance status, but patients in the control group were older (mean 59, SD 13 years vs mean 68 SD 13 years; *P*<.001) (Table 1).

**Table 1:** Participants, comorbidities, cancer type, performance status, and treatment.

Item			Intervention			Control		*P* value^a^
Age (years), mean (SD)			59 (13)			68 (13)		<.001
Gender female, n (%)			27 (54)			29 (58)		.69
**Comorbidities, n**								—^b^
	Diabetes			2			8	
	Chronic obstructive pulmonary disease			4			5	
	Ischemic heart disease			1			5	
**Cancer type, n (%)**								.36
	Breast			12 (24)			11 (22)	
	Bowel			13 (26)			9 (18)	
	Lung			6 (12)			15 (30)	
	Kidney			3 (6)			2 (4)	
	Rectal			3 (6)			2 (3)	
	Pancreatic			1 (2)			3 (6)	
	Prostate			2 (4)			2 (4)	
	Esophagus			2 (4)			2 (4)	
	Testicular			3 (6)			0 (0)	
	Ovarian			1 (2)			2 (4)	
	Rectal			3 (6)			0 (0)	
	Endometrial			2 (4)			0 (0)	
	Gastric			1 (2)			0 (0)	
	Leiomyosarcoma			0 (0)			1 (2)	
	Mesothelioma			0 (0)			1 (2)	
**Performance status, n**								.31
	0			20			16	
	1			22			21	
	2			5			9	
	3			0			2	
**Treatment, n**								.67
	Chemotherapy	45			45			
	Radio- and chemotherapy	3			2			
	Surgery and chemotherapy	1			1			
	Surgery	0			1			
	Best supportive care	1			1			

^a^Chi-square test.

^b^Not applicable.

### Checklist Utilization

Checklists were used 2882 times by the 50 patients in the intervention group, a median of 62 times per patient with the number of uses ranging from 13 to 102 times over the study period. App use resulted in no alert being generated on 2715 occasions, indicating no or no significant symptoms. On 167 (5.8%) occasions, actions were advised. There were 130 green alerts, 28 amber alerts, and 9 red alerts.

Usage by patients was 284 times in the first week, 347 times in the second week, and 228 times in the ninth week of participation.

Of the 50 nominated friends and family members, 31 used the checklists in the app to support their patient partner for a total of 318 times. Usage generated no alert on 267 occasions; in 28 instances, contact of a health care professional was advised. There were 18 amber alerts, 9 red, and 1 major alert.

Friends and family members used the app 77 times in the first week, 67 times in the second week, and 16 times in the ninth week.

Symptoms flagged by the checklists were, in order of frequency, exhaustion (102), nausea (23), fever (14), chest pain (13), sore mouth (13), diarrhea (11), pain (8), constipation (5), skin and eye complaints (4), pins and needles (3), mental health issues (2), visual disturbances (1), and urinary symptoms (1).

Logs completed by the acute oncology team indicated 23 patient calls in response to checklist items. Calls covered a broad range of topics, including technical advice (2 calls), reassurance (8 calls), and advice to admit (4 calls).

### Clinical Outcomes

Patients in the intervention group had 19 scheduled inpatient days, 40 unscheduled days in the hospital, and 32 unscheduled days in the hospital involving overnight stays. Patients in the control group had 2 scheduled inpatient days, 108 unscheduled days in the hospital, and 97 unscheduled days in the hospital involving overnight stays. There were 40 patients in the intervention group and 38 patients in the control group who spent no unscheduled time in the hospital. Patients in the intervention group required a third as many hospital days with overnight stay in comparison to the control group.

Patients and primary care practices requested information about appointments in primary care. In the first week after enrollment, patients in the intervention group saw their general practitioner 10 times, and patients from the control group 3 times. In the subsequent 3 weeks, patients from the intervention group saw a general practitioner 20 times, and patients in the control group saw a general practitioner 14 times. In the second month, patients from the intervention group saw a general practitioner 30 times, and patients from the control group had 15 visits.

### Anxiety and Depression

**Figure 3:**
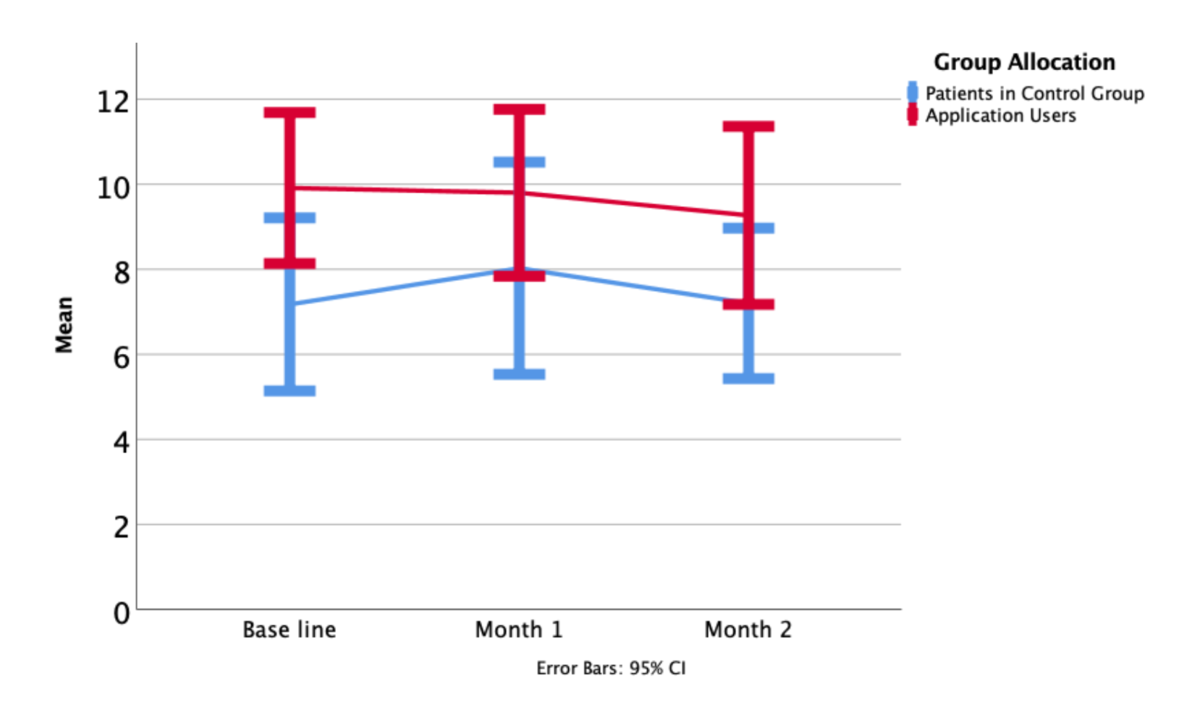
Hospital Anxiety & Depression Scale mean at baseline, one month, and at the end of the study period.

Patient experience was captured by standardized questionnaires and informal feedback from inside the application. A HADS score of 11 or more indicates clinically significant anxiety or depression. At baseline, the average HADS score was 7.9 (SD 7.2) in the control group and 10.2 (SD 6.1) in the intervention group (Figure 3). HADS scores of 11 or greater were observed in 14 patients in the control group and 26 patients in the intervention group. After one month, participants with a HADS score >11 had declined to 13 in the control group and 20 in the intervention group. At two months, 11 control patients and 18 intervention patients fulfilled the same criteria.

**Figure 4:**
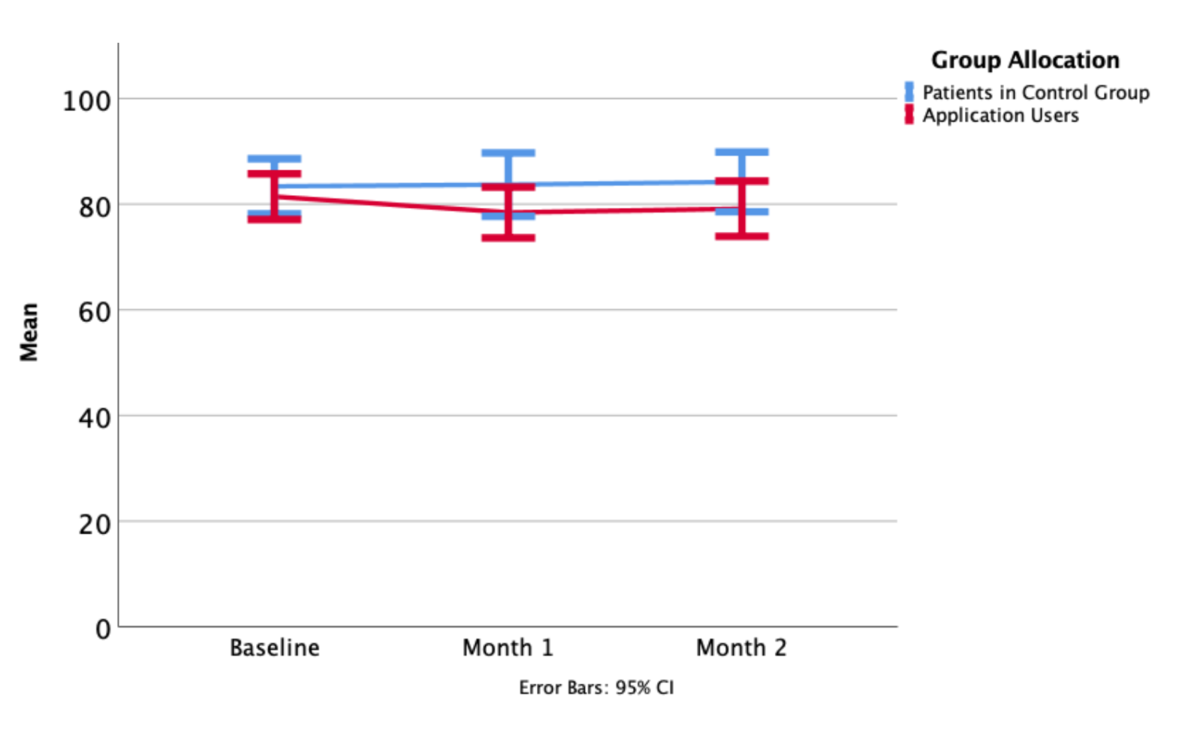
General version of the Functional Assessment of Cancer Therapy (FACT-G) overall means at baseline, one month, and at the end of the study period.

**Figure 5:**
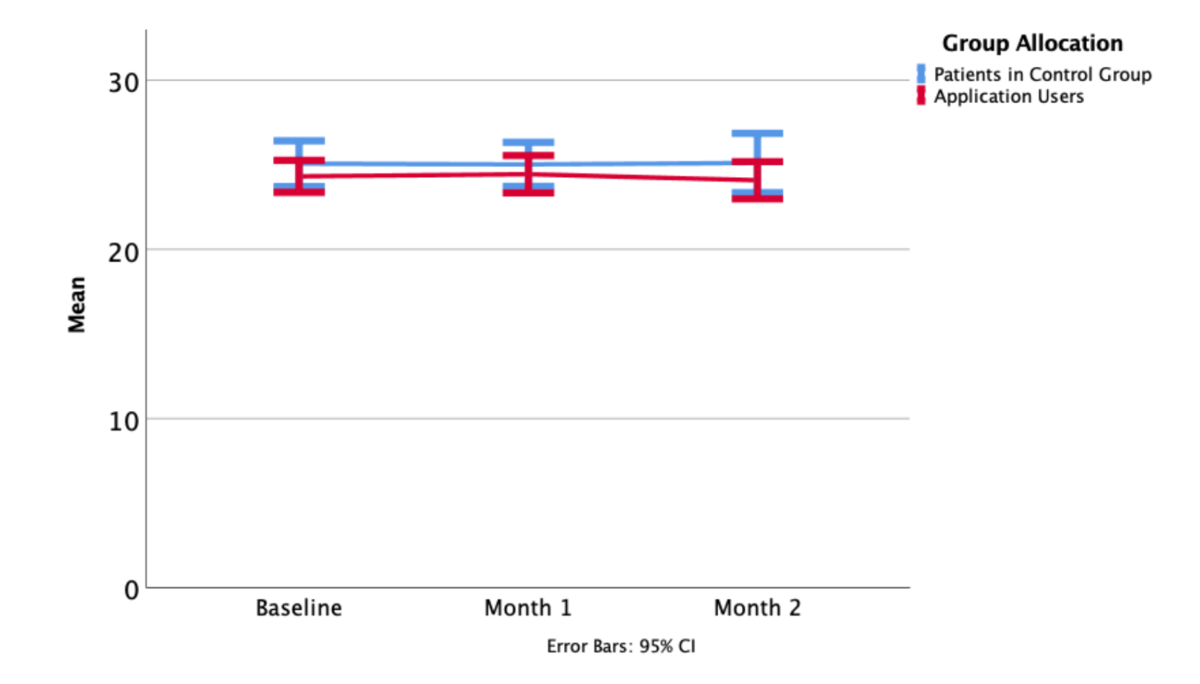
General version of the Functional Assessment of Cancer Therapy (FACT-G) Social and Family wellbeing subscore means at baseline, one month, and at the end of the study period.

**Figure 6:**
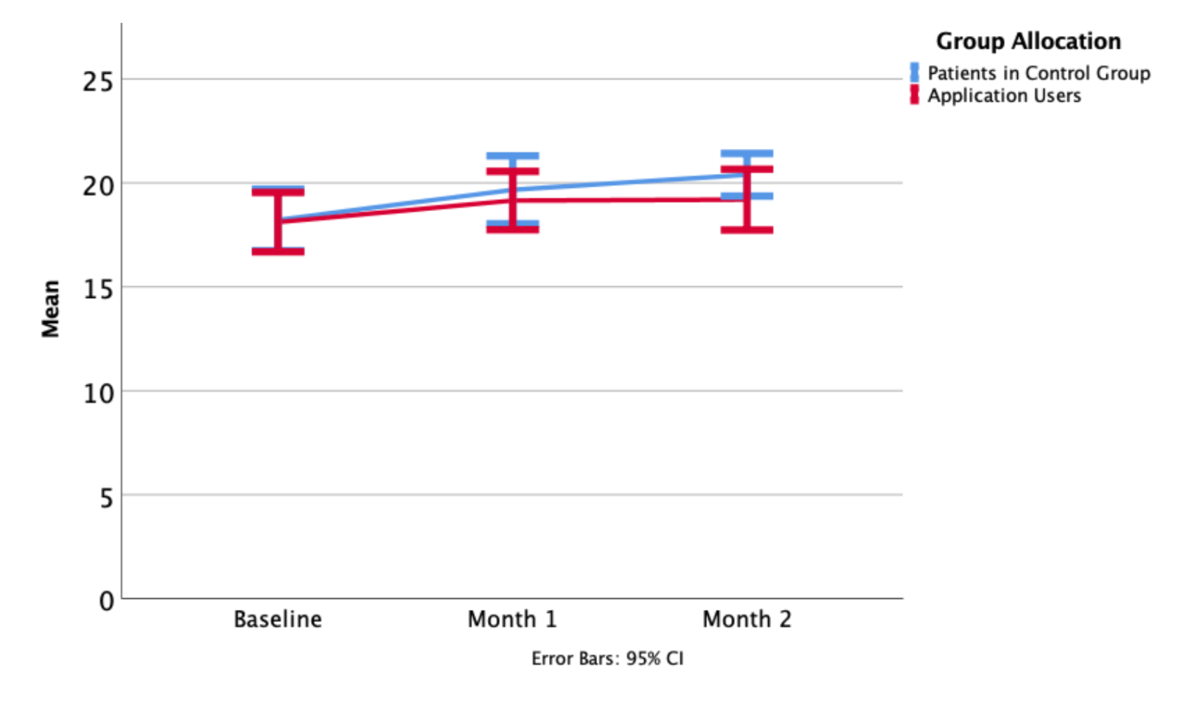
General version of the Functional Assessment of Cancer Therapy (FACT-G) Emotional wellbeing subscore means at baseline, one month, and at the end of the study period.

Values for the FACT-G were not significantly different at baseline, one month, or two months. Over the full duration of the study, 6 patients in the control group and 10 patients in the intervention group improved by more than 10% over their baseline (Figures 4-6).

### Feedback From Patients, Friends, and Families

Structured feedback was submitted from within the application by 48 patients and 25 family members and friends (Table 2). Mean ratings on a Likert scale with values from 1 (strongly disagree) to 5 (strongly agree) to the question “I feel safer with the checklist” was 4.27 (SD 0.87) for patients and 4.55 (SD 0.65) for family members and friends. The question “The link to a health care professional is helpful” yielded mean ratings of 4.61 (SD 0.74) for patients and 4.75 (SD 1.35) for family members and friends.

**Table 2:** Structured feedback using Likert scales with values from 1 (strongly disagree) to 5 (strongly agree).

Feedback question	Patients (all)	Family and friends (all)	Patients		
			First assessment	Last assessment	Delta
The information in the app is helpful, mean	4.32	4.2	4.07	4.45	0.38
The link to a friend or relative is helpful, mean	4.48	4.41	4.42	4.27	–0.15
The link to a health care professional is helpful, mean	4.61	4.75	4.55	4.66	0.11
I feel safer with the checklist, mean	4.27	4.55	4.17	4.41	0.24

## Discussion

### Principal Findings

With the KeepMeSafe application, patients and their families and friends were able to use a smartphone app to work through a list of common complications of cancer and systemic therapies. We demonstrated the feasibility of assistance by members of the patients’ social network at times when patients felt unable to complete the checklist themselves. To our knowledge, this is the first time that patients and members of their social network have been deployed as redundant parts of a safety system.

Patients were only able to participate if they owned a smartphone. This limitation might exclude some patients, but the percentage of people actively using smartphones in the UK in 2019 was 82.9%, the highest in the world [23]. The limited size of our study and the fact that the intervention and control groups were not randomized or matched means that the study does not allow conclusions about clinical outcomes, effectiveness, or efficiency.

Patients experience higher levels of anxiety and depression than the general population [22] though consistent with other contemporaneous cohorts of people who have cancer in the UK [24].

In a review of the literature of clinical trials involving mobile health apps, we found 17 studies of between 12 and 2352 patients [25]. Smartphone apps or internet portals primarily collected data on clinical symptoms or activity data with some improvement in patient-reported outcome measures. The authors found limited evidence for effects on mortality or cancer-related morbidity, including complications and health-economic outcomes. Many studies did not report on app usage. Only a few studies have reported improvements in quality of life [26,27]. App for monitoring pain and linked to the ability to escalate to a clinician might lead to improved symptom control [28]. Recruitment rates of 50% in our study are comparable to other trials in this field [29].

We collected data of indicative health-service usage by reporting days spent in the hospital and appointments with primary care physicians. We observed trends towards increased usage of primary care appointments and decreased usage of hospital days for unscheduled admissions in the younger intervention group.

App usage was high and comparable with other high-quality applications [30]. Patients and their buddies reported satisfaction with the information in the app and its links to health care staff and reported feeling safer with the application. It is difficult to say that patients felt more empowered to reach out to health care staff (or that the app encouraged them to do so), given that the majority of contacts were initiated by nursing staff. At least eight of these contacts received telephone advice, however, and it might be inferred that having easy access to health care staff in this way reduced the burden on primary care services.

Limitations identified in the literature review were addressed by measuring app usage and validated clinical outcome measures and surrogates for health economic measures, albeit in a non-randomized single-center study. Many mobile health apps are designed for single diseases [31-33] or use generic metrics such as physical activity [34], with only a few applications reporting patient outcomes [35]. By using a content management system as the underlying architecture, we enabled agile, modular development for future expansion to rarer complications, tailoring to different cancers, individualized treatment regimes, and patient preferences. While this study was limited in scope to proof-of-concept, it has generated the methodology for larger trials powered to demonstrate improvements in patient-centric outcomes.

The study demonstrates that patients and those close to them can take an active part in a redundant safety system. Technology can facilitate laypersons to undertake some of the safety-critical screening functions that are normally undertaken by nurses based on the UKONS clinical checklists.

Real-time response to alerts would require 24/7 cover of staff who are familiar with diseases and treatment modalities. Scale-up of usage, including utilization for follow-up of patients with cancer, would require limited investment into the soft-ware platform but reliable investment into the teams that support cancer services locally and nationally.

This application may be a useful tool in aiding patients to access early and appropriate acute cancer care. It may also have a role in supporting ambulatory outpatient management of presentations suitable for this model of care.

Future research will have to tease out the effect size in multiple settings. The number of friends and family members forming a safety network for patients may be relevant for the effect-size; several safety partners might support patients better than just a single partner.

Ways to strengthen ownership and activation of patients in future versions of the application might include incentives for usage or link to continuous monitoring with wearable sensors to supplement patient-reported symptoms with quantitative measures of risk [36].

The hypothesis of the checklist application that remains to be tested in larger trials is that usage of electronic checklists tailored to the needs of patients with cancer will improve reliability and timeliness of engagement with their multi-disciplinary team.

We hope to affect patients with cancer positively by first facilitating safer care: complications are, in large part, predictable. Checklists allow patients to be actively involved in the prevention of adverse events. Modular redundancy of safety-critical processes is a key mechanism to provide safe and stable systems in other industries [10]. The usage of checklists by multiple partners should ultimately lead to a testable reduction in preventable adverse outcomes. Lastly, we believe in the value of greater autonomy of patients through participation. Access to safety-critical information in a personalized and context-specific way is key for patient activation [37]. We fully expect that this will also improve resilience to acute complications [38].

### Conclusions

We coproduced a checklist application for smartphones with cancer patients, their friends, and families and demonstrated proof of concept as a networked and scalable safety intervention.

It is feasible to enable patients undergoing treatment for cancer to contribute to their own safety in recognizing complications of cancer and their therapy. To assess the impact on clinical outcomes requires larger randomized trials but utilizing such applications may form a key aspect of future acute cancer care.

## Abbreviations

FACT-G: General version of the Functional Assessment of Cancer Therapy
UKONS: United Kingdom Oncology Nursing Society
