# Correction: Checklists for Complications During Systemic Cancer Treatment Shared by Patients, Friends, and Health Care Professionals: Prospective Interventional Cohort Study

**DOI:** 10.2196/24816

**Published:** 2020-10-07

**Authors:** Helen V Jones, Harry Smith, Tim Cooksley, Philippa Jones, Toby Woolley, Derick Gwyn Murdoch, Dafydd Thomas, Betty Foster, Valerie Wakefield, Pasquale Innominato, Anna Mullard, Niladri Ghosal, Christian Subbe

**Affiliations:** 1 Ysbyty Gwynedd Penrhosgarnedd Bangor United Kingdom; 2 School of Medicine Cardiff Univeristy Cardiff United Kingdom; 3 The Christie Manchester United Kingdom; 4 UK Oncology Nursing Society Marlow United Kingdom; 5 Galactig Lôn Cae Ffynnon Cibyn Caernarfon United Kingdom; 6 North Wales Cancer Forum Bangor United Kingdom; 7 Cancer Chronotherapy Team Warwick Medical School Coventry United Kingdom; 8 European Laboratory U935, Institut National de la Santé et de la Recherche Médicale (INSERM) Paris-Saclay University Villejuif France; 9 North Wales Cancer Centre Rhyl United Kingdom; 10 School of Medical Sciences Bangor University Bangor United Kingdom

In “Checklists for Complications During Systemic Cancer Treatment Shared by Patients, Friends, and Health Care Professionals: Prospective Interventional Cohort Study” (JMIR Mhealth Uhealth 2020;8(9):e19225) the authors noted one error.

Author Derick Gwyn Murdoch was incorrectly listed with given names “Derick Gwyn” and surname “Murdoch”, and listed in the article citation as “Murdoch DG.” This was incorrect and has been changed to given name “Derick” and surname “Gwyn Murdoch”, with citation “Gwyn Murdoch D”.

The correction will appear in the online version of the paper on the JMIR Publications website on October 7, 2020, together with the publication of this correction notice. Because this was made after submission to PubMed, PubMed Central, and other full-text repositories, the corrected article has also been resubmitted to those repositories.

